# Concurrent variations of the left renal and gonadal vessels: a case report

**DOI:** 10.1590/1677-5449.202500902

**Published:** 2026-03-23

**Authors:** Surekha Devadasa Shetty, Mohandas Rao Kappettu Gadahad, Ashwini Aithal Padur, Naveen Kumar, Satheesha Badagabettu Nayak

**Affiliations:** 1 Manipal Academy of Higher Education, Manipal, India.; 2 RAK Medical & Health Sciences University, Ras Al Khaimah College of Medical Sciences, Ras Al Khaimah, United Arab Emirates.

**Keywords:** case report, renal artery, renal vein, testicular vein, testicular artery, relato de caso, artéria renal, veia renal, veia testicular, artéria testicular

## Abstract

A thorough knowledge of vascular variations in the vicinity of the kidney is essential due to the increased number of kidney transplants, more so on the left side because the donor’s left kidney is preferred for transplantation. We found multiple vascular variations in the vicinity of the left kidney, which were noted during routine dissection classes. The left renal artery arose from the abdominal aorta and supplied the left kidney in a usual manner. There was an additional left renal artery, which took its origin from the abdominal aorta, below the origin of the inferior mesenteric artery. This artery was accompanied by an additional renal vein, which drained into the left common iliac vein. On the left side, there were two testicular veins. Knowledge of these abnormal vessels can be useful during radiological and surgical procedures in cases of nephrectomies, renal tumors, transplants, and renal vascular illnesses.

## Resumo

Um conhecimento aprofundado das variações vasculares na região adjacente ao rim é essencial, considerando o aumento do número de transplantes renais, especialmente no lado esquerdo, uma vez que o rim esquerdo do doador é preferido para o transplante. Foram identificadas múltiplas variações vasculares na região do rim esquerdo, observadas durante aulas rotineiras de dissecação. A artéria renal esquerda originava-se da aorta abdominal e irrigava o rim esquerdo de maneira habitual. Além disso, havia uma artéria renal esquerda adicional, que se originava da aorta abdominal abaixo da origem da artéria mesentérica inferior. Essa artéria era acompanhada por uma veia renal adicional, que drenava para a veia ilíaca comum esquerda. No lado esquerdo, observou-se a presença de duas veias testiculares. O conhecimento dessas variações vasculares é útil durante procedimentos radiológicos e cirúrgicos, especialmente em casos de nefrectomias, tumores renais, transplantes e doenças vasculares renais.

## INTRODUCTION

The renal arteries are a pair of lateral branches of the abdominal aorta arising just below the level of the origin of the superior mesenteric artery.^1^ Variations in number and branching of renal arteries are common.^2,3^ Renal artery variations have been classified as aberrant, supplementary, and accessory, among other terms.^1^ Accessory renal arteries are frequently seen, especially on the left side.^4^ They usually enter above or below the renal hilum.^5^ Supernumerary renal arteries are of two types: polar or accessory hilar type. Polar type arteries enter the kidney through the pole without passing through the hilum, whereas accessory hilar type arteries enter the kidney after passing through the hilum.^6^ Various developmental positions of the kidney are one of the main reasons for renal arteries abnormalities.^7^

The renal veins lie anterior to the renal artery and open into the inferior vena cava at the level of the 1st and 2nd lumbar vertebrae. The right renal vein is a short vessel and does not receive any tributaries except from the right kidney. The left renal vein crosses anterior to the aorta to open into the left lateral aspect of inferior vena cava and is three times longer than the right. It usually receives two tributaries, the left suprarenal vein and the left gonadal vein.^5^

The testicular artery is a branch of the abdominal aorta, given off at the level of the 2nd lumbar vertebra below the renal artery. The testicular artery may show variations at its origin or may be absent. One or both of these arteries may arise from the renal artery, suprarenal artery, or lumbar artery. The testicular veins accompany the arteries.^8^ Congenital variations of the testicular vein include variations in its course, areas of drainage, and termination.^9^

Here, we present a case of concurrent variations of the left renal and gonadal vessels. This variation could predispose a person to varicocele and infertility. Urologists and radiologists may find this case useful in their practice.

## CASE REPORT

**Figure 1:**
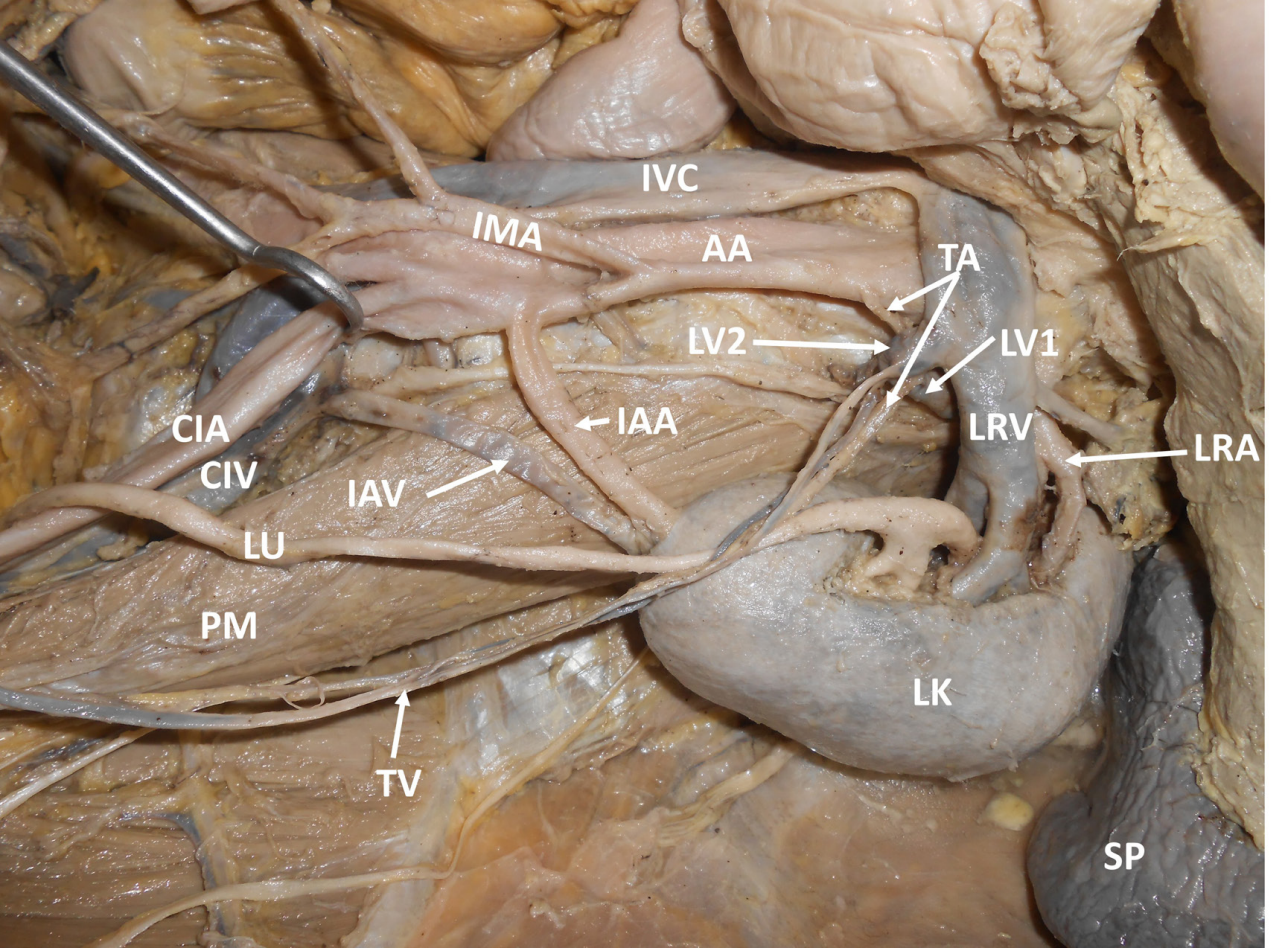
Dissection of the upper abdomen, showing the multiple vascular variations. IVC – inferior vena cava; AA – abdominal aorta; IMA – inferior mesenteric artery; LRV – left renal vein; LRA – left renal artery; SP – spleen; LK – left kidney; LU – left ureter; IAA – inferior accessory artery; IAV – inferior accessory vein; LV1 – first lumbar vein; LV2 – second lumbar vein; TA – testicular artery; TV –testicular veins; PM – psoas major; CIV – left common iliac vein; CIA – left common iliac artery.

**Figure 2:**
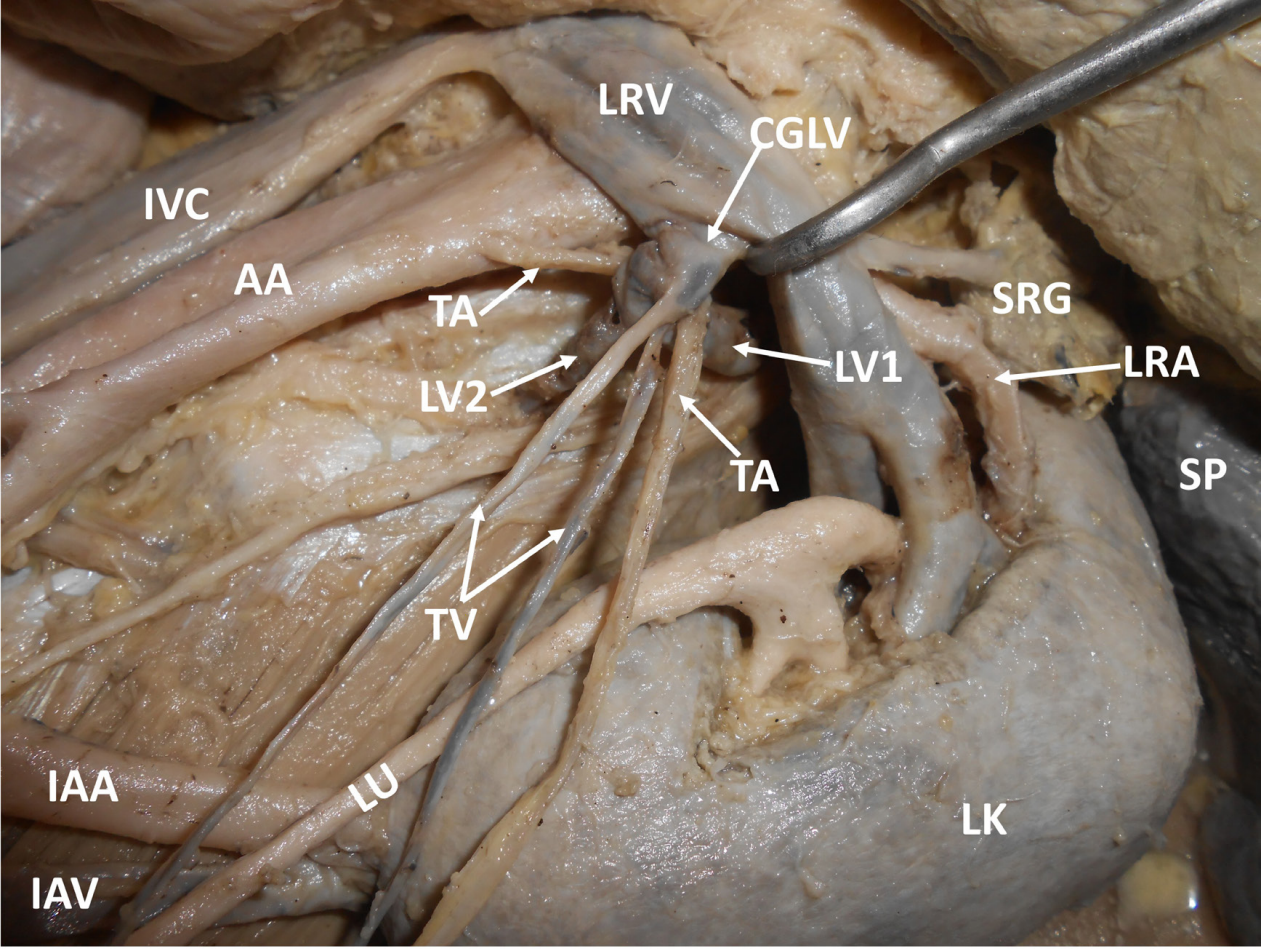
Closer view of the multiple vascular variations. IVC – inferior vena cava; AA – abdominal aorta; LRV – left renal vein; LRA – left renal artery; SP – spleen; LK – left kidney; LU – left ureter; IAA – inferior accessory artery; IAV – inferior accessory vein; CGLV – common gonado-lumbar vein; LV1 – first lumbar vein; LV2 – second lumbar vein; TA – testicular artery; TV –testicular veins; SRG – suprarenal gland.

During dissection classes for medical undergraduates, we witnessed multiple vascular variations in relation to the left kidney. The variations were unilateral and were observed in a South Indian adult male cadaver. The left kidney was supplied by the left renal artery, arising as usual from the abdominal aorta, and was drained by the left renal vein into the inferior vena cava. Apart from this, the left kidney was also supplied by an aberrant renal artery arising from the abdominal aorta, one inch below the origin of the inferior mesenteric artery. This artery entered the kidney through its inferior pole. This artery was accompanied by an aberrant renal vein, which drained into the left common iliac vein. There were two testicular veins on the left side. About 1cm below the left renal vein, these two veins united with the first and second lumbar veins to form a ‘common gonado-lumbar vein’. The common gonado-lumbar vein drained into the left renal vein. Further, the left testicular artery arose from the abdominal aorta, ascended upwards and hooked around the junction of the first lumbar vein with the common gonado-lumbar vein. The further course of the testicular artery was normal. These variations are shown in Figures 1 and 2. Ethics committee clearance was obtained for the study. This study complies with the Helsinki Declaration and with local ethical guidelines. We have obtained all appropriate consent forms and ethics committee clearance for the use of cadavers in this study. No patient data were used in the study.

## DISCUSSION

Other than the main renal artery, any artery arising from the abdominal aorta and entering the kidney should be named an accessory renal artery and any renal artery that arises from a source other than the aorta and enters the kidney should be called an aberrant renal artery.^10^ When aberrant renal arteries enter the upper or lower poles of the kidney, they are named polar arteries.^11^ Accessory renal arteries are end arteries and if an accessory renal artery is ligated, the part of the kidney it supplies becomes ischemic.^4^ Abnormal renal arteries penetrate the substance of the kidney rather than entering its hilum to supply it. The currently observed variant renal artery, entering the left kidney through the inferior pole should be called by all three names, as accessory renal artery, aberrant renal artery, and inferior polar artery. Inferior polar arteries have been implicated as an etiological factor in a form of hydronephrosis correctable by surgery.^12^

Many authors have reported the presence of aberrant renal arteries. They happened to enter the kidney as either upper or lower polar arteries. Short polar arteries can enhance the degree of difficulty during surgical procedures.^13,14^

The left renal vein normally drains into the inferior vena cava, after passing anterior to the abdominal aorta. There are also reports of lumbar veins draining into the left renal vein. Recently, a case of renal vein variation was reported in which left suprarenal, ovarian, and lumbar veins drained into a single left renal vein which then drained into the left common iliac vein.^15^ In another case, there were two left renal veins, one of which retroaortically drained into the inferior vena cava, while the other drained into the left common iliac vein.^16^ A retroaortic course of the left renal vein is relatively rare.^17^ Very rarely, the left renal vein receives the left gonadal vein indirectly through the kidney. A case has also been reported in which the left testicular vein penetrated the lower pole of the kidney before opening into the renal vein.^18^ In some cases, the inferior pole of the kidney drains into the contralateral common iliac vein through a separate vein.^19^

Testicular veins can be absent, double, triple, or multiple.^20,21^ Variations in the terminations of testicular veins have also been reported. The right testicular vein might drain into the right renal vein, an accessory renal vein, or the lower part of the inferior vena cava.^22^ The left testicular artery may arch around the left renal artery or renal vein before it descends.^23^

### Possible embryologic basis of the variation

The definitive left renal vein develops from the left mesonephric vein, left subcardinal vein, and intersubcardinal anastomosis. The part of the subcardinal vein above the renal vein forms the left suprarenal vein and the part below forms the gonadal vein. The subcardinal vein communicates with nearby venous channels like the supracardinal veins and azygos line of veins and eventually disappears.^7^ Formation of the gonado-lumbar vein in the current case could be due to the failure of the communications to disappear. The left testicular artery’s looping course around the gonado-lumbar vein could also be due to the union of lumbar veins with the testicular veins. It is very simple to understand the presence of the aberrant inferior polar artery and vein. The kidney begins its development in the pelvic cavity and later ascends. While in the pelvis, it derives its blood supply from the iliac vessels. Later, as it ascends, it receives blood supply from the aorta and drains into the inferior vena cava. During the process of ascent, the pelvic and lower lumbar vessels supplying it degenerate and disappear. In the current case, the presence of aberrant vessels is due to their failure to disappear.

### Clinical importance of the case

The current pattern of multiple variations could lead to the development of left sided varicocele. As such, varicoceles are more common on the left side. In the current case, the abnormal hooking of the left testicular artery around the gonado-lumbar vein could alter the hemodynamics of the left testis leading to infertility. Knowledge of the above-mentioned variations could be useful during renal transplant surgeries, therapeutic embolization of testicular veins, and abdominal radiological procedures.

## CONCLUSION

Knowledge of renal and gonadal vascular variations is essential for diagnostic, endovascular, and operative procedures involving the abdomen. This report highlights the presence of an aberrant left renal artery and a variant type of termination of the left testicular vein. The unique feature of this case report is the formation of a gonado-lumbar vein by the union of two left testicular veins and the first and second lumbar veins. There are no previous reports of such a termination of the left testicular vein.

## Data Availability

Data sharing does not apply to this article, as no data were generated or analyzed.

## References

[1] Standring S (2016). Gray’s Anatomy: the anatomical basis of clinical practice..

[2] Asala S, Chaudhary SC, Masumbuko-Kahamba N, Bidmos M (2001). Anatomical variations in the human testicular blood vessels. Ann Anat.

[3] Nayak SB, Shetty SD, Ravindra S (2014). Eight prehilar branches of the right renal artery. Anat Cell Biol.

[4] Satyapal KS, Haffejee AA, Singh B, Ramsaroop L, Robbs JV, Kalideen JM (2001). Additional renal arteries; Incidence and morphometry. Surg Radiol Anat.

[5] William PL, Bannister LH, Berry MM, Gray H (1995). Gray’s Anatomy.

[6] Ozkan U, Oguzkurt L, Tercan F, Kizilkilic O, Koc Z, Koca N (2006). Renal artery origins and variations: angiographic evaluation of 855 consecutive patients. Diagn Interv Radiol.

[7] Moore KL, Per Sand TVN (2015). The developing human..

[8] Bergman RA, Cassell MD, Sahinoglu K, Heidger PM (1992). Human doubled renal and testicular arteries. Ann Anat.

[9] Asala S, Chaudhary SC, Masumbuko-Kahamba N, Bidmos M (2001). Anatomical variations in the human testicular blood vessels. Ann Anat.

[10] Graves FT (1956). The aberrant renal artery. J Anat.

[11] Khamanarong K, Prachaney P, Utraravichien A, Tong-Un T, Sripaoraya LX (2004). Anatomy of renal arterial supply. Clin Anat.

[12] Bergman RA, Thomson SA, Afifi AK, Saadeh FA (1998). Compendium of human anatomic variation..

[13] Patil SJ, Mishra S (2013). Inferior renal polar artery and its surgical importance. OA Anatomy.

[14] Chatzizacharias NA, Muthusami ASR, Sullivan M, Sinha S, Brockmann J (2010). Use of gonadal vein interposition graft for implantation of polar artery in live donor renal transplantation. Transplantation.

[15] Kaabneh AB, Haddad SE, Hammouri FA, Omari AY, Abdadayem MK (2017). A single left renal vein draining into the common iliac vein. Indian J Urol.

[16] Kawai K, Tanaka T, Watanabe T (2016). A rare anomaly of left renal vein drainage into the left common iliac vein: A case report. Int J Surg Case Rep.

[17] Sonawane GB, Moorthy KH, Pillai BS (2020). Newer variants of retroaortic left renal vein. Indian J Urol.

[18] Rosalino UAC, Latorre GC, Pinto AC, Toscano MP (2011). Uncommon drainage of the gonadal vein: case report. J Morphol Sci..

[19] Pranav Divakaran P, Robert MJ, Perera M, Pais A, Pretorius CF (2016). Congenital contralateral venous drainage of a right ectopic kidney to left common iliac vein: a rare case report. Eur J Anat.

[20] Nayak SB (2019). Five Veins at the Deep Inguinal Ring. Can they Reduce the Chances of Indirect Inguinal Hernia and Increase the Chances of Varicocele?. Int J Morphol.

[21] Nayak SB, Kodimajalu VS (2020). Triple right testicular veins and their variant termination and communications. Heliyon.

[22] Asala S, Chaudhary SC, Masumbuko-Kahamba N, Bidmos M (2001). Anatomical variations in the human testicular blood vessels. Ann Anat.

[23] Nayak SB (2007). Abnormal course of left testicular artery in relation to an abnormal left renal vein – a case report. Kathmandu Univ Med J.

